# Supplementary material for: Automated detection and segmentation of non-small cell lung cancer computed tomography images
Source: Nat Commun. 2022 Jun 14;13:3423. doi: 10.1038/s41467-022-30841-3 (PMC9198097; doi:10.1038/s41467-022-30841-3)
Supplement: Supplementary file 1 — Supplementary Information [file 41467_2022_30841_MOESM1_ESM.pdf]

Supplementary materials for "Automated detection and segmentation of non-small cell lung cancer computed tomography images"

Supplementary Table(s)

Supplementary Table 1: Python packages

Purpose	Package name	Version
**Pre-processing, data handling:**		
	os, re, random, time	python standard library
	psutil	5.6.1
	numpy	1.16.2
	pandas	0.25.1
	pydicom	1.3.0
	opencv-python	4.1.0.25
	SimpleITK	1.2.0
	scikit-image	0.14.2
	scipy	1.2.1
	scikit-learn	0.23.2
	Tqdm	4.40.2
**Deep Learning:**		
	keras	2.2.4
	tensorflow-gpu	1.15.0
	livelossplot	0.4.1
**Visualization:**		
	matplotlib	3.0.3
	seaborn	0.9.0
**In- house developed packages:**		
	precision medicine toolbox (https://github.com/primakov/precision-medicine-toolbox)	1.0

Supplementary Table 2: Datasets population description

Dataset	Histology (Adeno-carcinoma %)	Age (range, years)	Sex (Male %)	T stage (T4 %)	N stage (N1,2%)	M stage (M1 %)	Chemo-therapy (Yes %)	Radiation (Yes %)
Maastro-CT-Lung-1	12.1%	33-92	66.7%	27.7%	38.9%	0.7%	53.5%	46.5%
UCL-CT-Lung	49.1%	44-84	82.5%	26.3%	31.6%	0.0%	73.7%	21.1%
UCSF-CT-Lung	61.3%	46-92	46.0%	0.0%	8.0%	0.0%	NA	100%
AZHDU Lung	68.7%	27-81	34.4%	0.0%	0.1%	0.0%	NA	NA
Stanford Lung	81.5%	24-87	64%	3.3%	15.6%	2.4%	23.2%	7.6%
TCIA-CT-Lung-3	37.1%	NA	67.4%	3.4%	28.0%	5.6%	NA	NA
The Maastro interobserver reproducibility test	43.5%	40-82	56.5%	8.7%	78.3%	0.0%	NA	NA
Radboud Lung 2	29.6%	NA	NA	22.9%	52.6%	0.0%	NA	100%
MUMC/Heerlen lung	38.6%	32-93	59.2%	27.1%	40.8%	5.9%	19.6%	NA

Supplementary Table 3: Univariate Cox regression C-index (CI), hazard ratio (HR), p-value. P-values were calculated using Wald test.

Dataset,Variable,Segmentation	CI	HR	p-value
Maastro-CT-Lung1, Volume, Manual	0.582	1.002	<0.0001
Maastro-CT-Lung1, Volume, Automated	0.597	1.003	<0.0001
Maastro-CT-Lung1, RECIST, Manual	0.581	1.008	<0.0001
Maastro-CT-Lung1, RECIST, Automated	0.607	1.007	<0.0001
Stanford Lung, Volume, Manual	0.643	1.003	0.108
Stanford Lung, Volume, Automated	0.638	1.003	0.093
Stanford Lung, RECIST, Manual	0.639	1.021	0.002
Stanford Lung, RECIST, Automated	0.617	1.006	0.046

Supplementary Figures

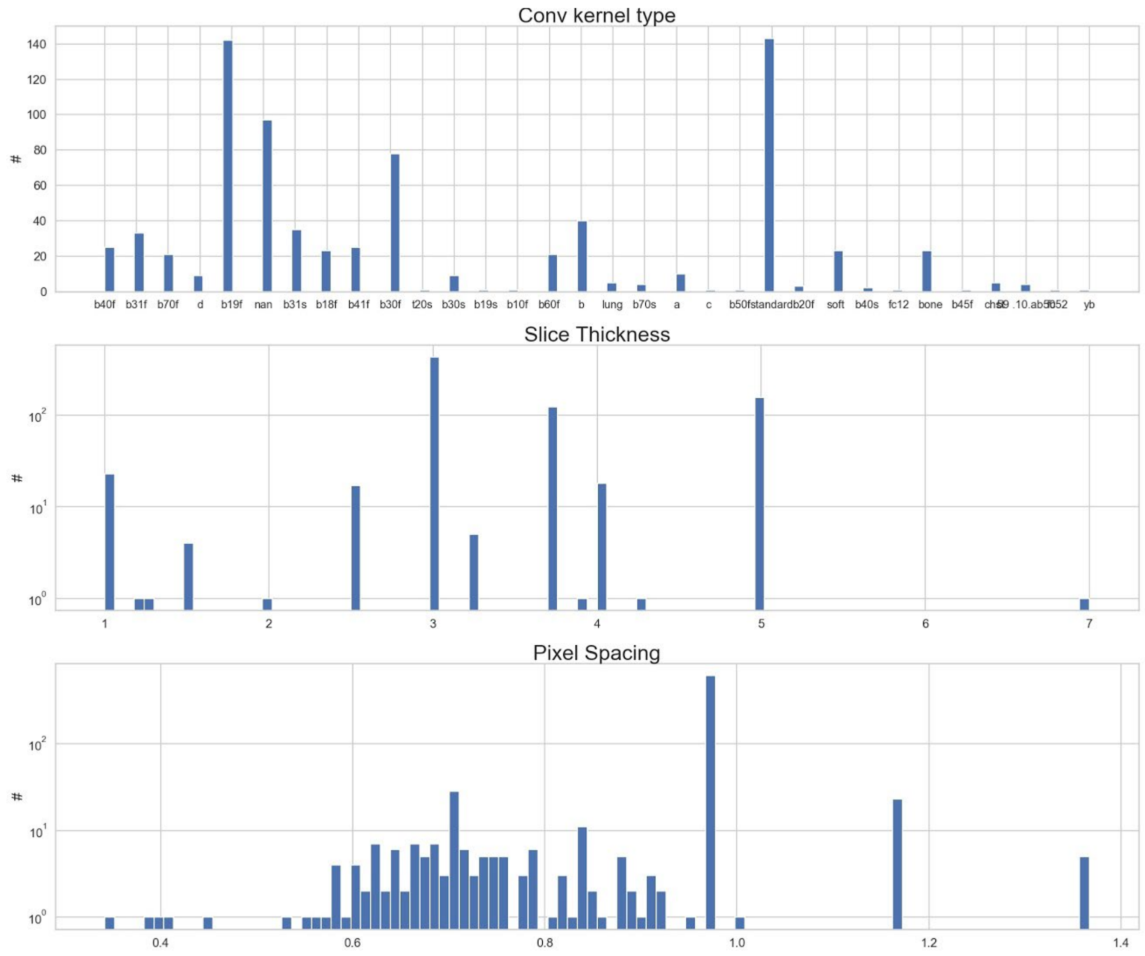

Fig. 1: Variability of CT reconstruction parameters in the training dataset.
Distributions of various convolutional kernels, slice thickness and pixel spacing parameters in the training dataset.

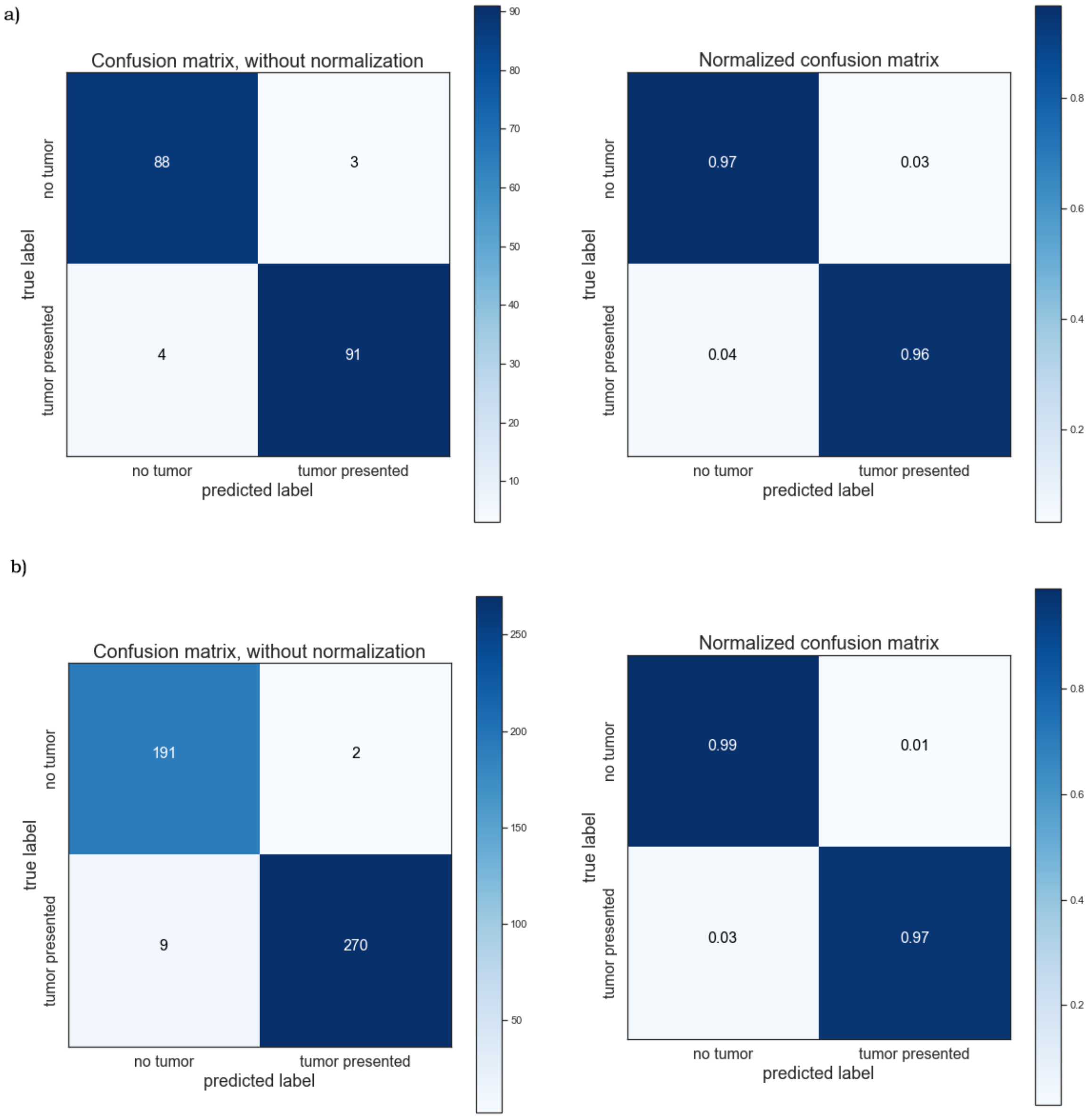

Fig. 2: Lung-vice quantitative detection performance.
a) test dataset containing 93 NSCLC independent CT scans. **b)** external validation dataset of 236 independent NSCLC CT scans. From left to right: original confusion matrix, normalized confusion matrix. Coloring of the matrix without normalization is obtained by using direct counts and for normalized matrix by normalizing absolute numbers to row sums.

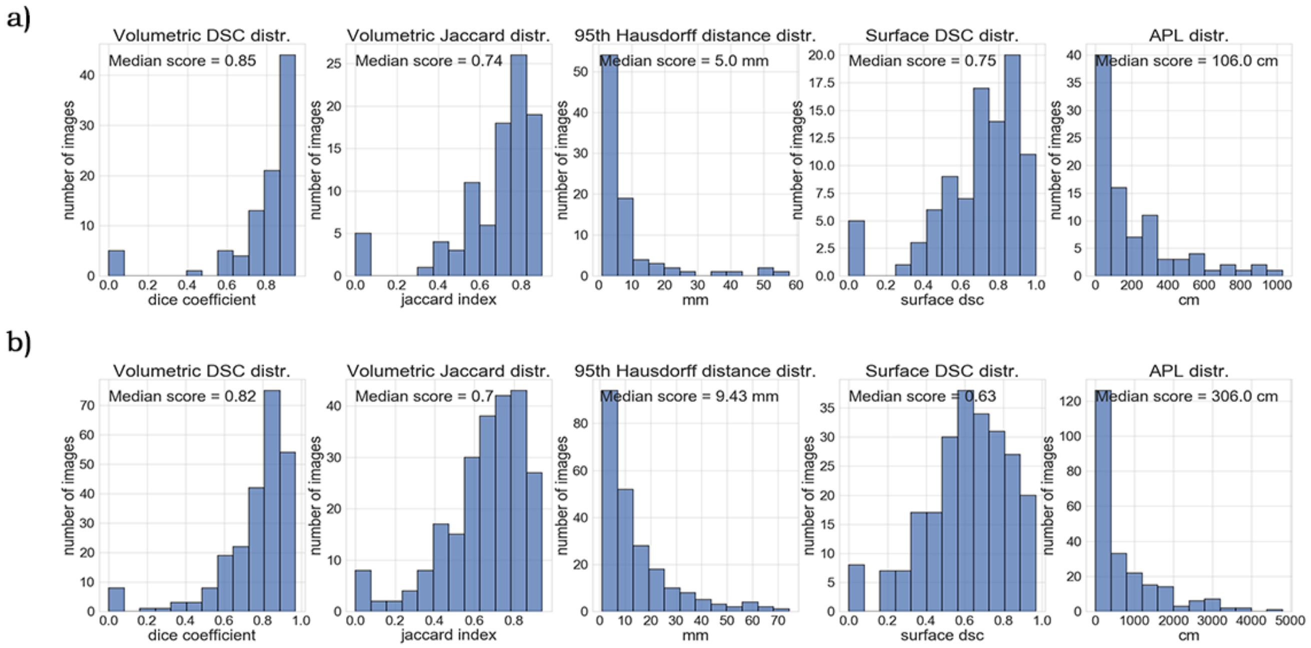

Fig. 3: Quantitative segmentation performance. Quantitative segmentation performance evaluated with: volumetric dice similarity coefficient (DSC), Volumetric Jaccard index (Ji), Robust Hausdorff distance (H95th), Surface DICE (Surface DSC), Added Path Length (APL). **a)** test dataset containing 93 NSCLC independent CT scans. **b)** external validation dataset of 236 independent NSCLC CT scans.

a) b)

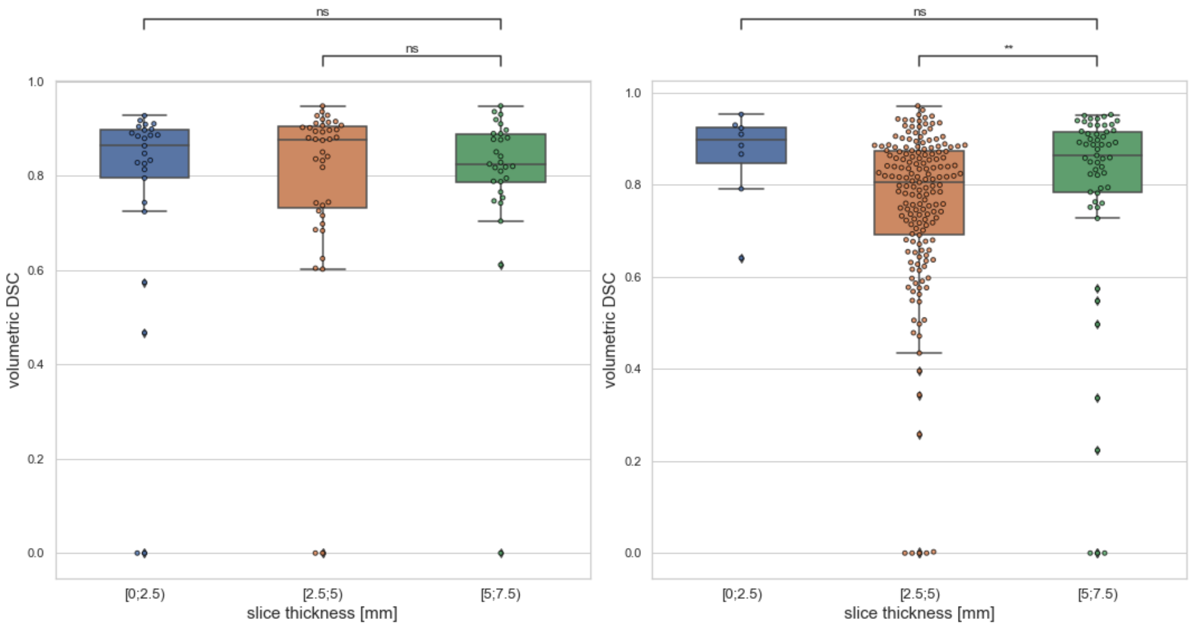

Fig. 4: Quantitative segmentation performance in regards to CT slice thickness. Quantitative segmentation performance is measured with volumetric dice similarity coefficient (DSC). Data are presented as box plots with overlaid swarm plot, where boxes are representing inter quartile range (IQR), extending from Q1 to Q3 and centered on the median value. Upper whiskers represent the highest data point that is less than Q3 + 1.5 × IQR. Lower whiskers represent the smallest data point that is greater than Q1 − 1.5 × IQR. Data points outside whiskers considered as outliers. P values were calculated using a two-sided Mann-Whitney-Wilcoxon test with Bonferroni correction and referred as follows: "ns" on the plot refers to the p-value in the range: $5.00e\text{-}02 < p <= 1.00e+00$; ** refers to the p-value in the range: $1.00e\text{-}03 < p <= 1.00e\text{-}02$. The exact p values reported in the order from the top to the bottom as they displayed on the figures. Corresponding p-values for the left figure: 1.000e+00, 1.000e+00; corresponding p values for the right figure: 7.832e-01, 2.629e-03.
a) model performance on the test dataset containing 93 NSCLC independent CT scans. **b)** model performance based on the external validation dataset of 236 independent NSCLC CT scans.

a) b)

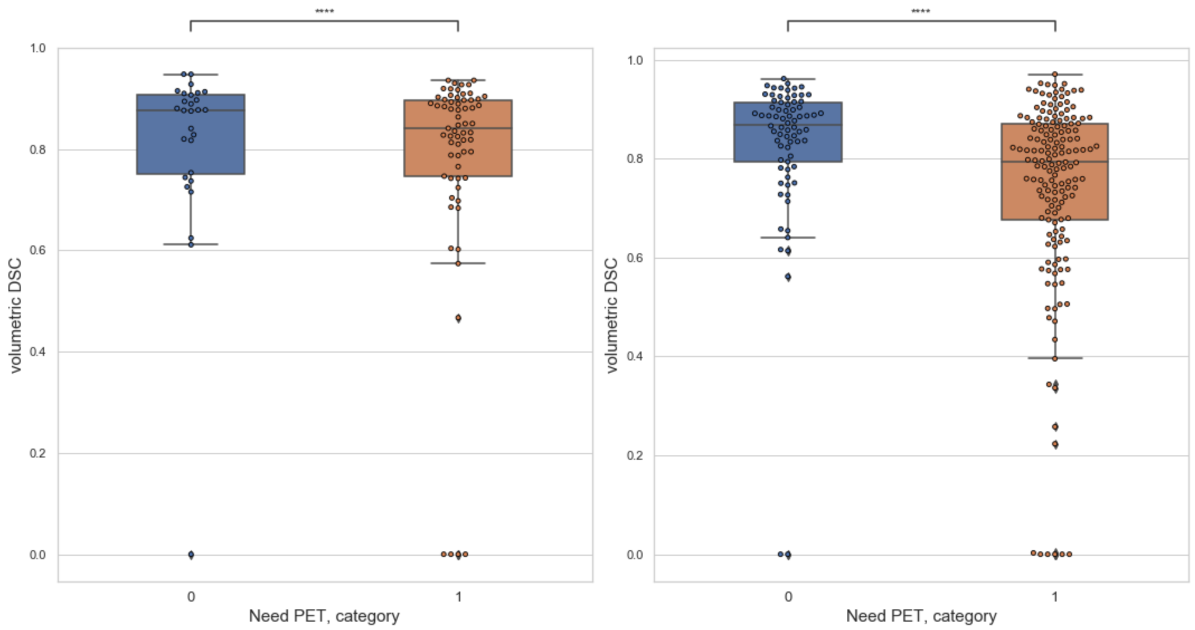

Fig. 5: Quantitative segmentation performance in regards to tumor complexity label. Quantitative segmentation performance is measured with volumetric dice similarity coefficient (DSC). Tumor complexity label is defined as follows 1: PET needed to produce segmentation, 0: No PET needed to produce segmentation. Data are presented as box plots with overlaid swarm plot, where boxes are representing inter quartile range (IQR), extending from Q1 to Q3 and centered on the median value. Upper whiskers represent the highest data point that is less than Q3 + 1.5 × IQR. Lower whiskers represent the smallest data point that is greater than Q1 − 1.5 × IQR. Data points outside whiskers considered as outliers. P-values were calculated using a two-sided Mann-Whitney-Wilcoxon test with Bonferroni correction and referred as follows: **** refers to the p-value in the range: p <= 1.00e-04. **a)** test dataset containing 93 NSCLC independent CT scans; p-value is 0.000e+00. **b)** external validation dataset of 236 independent NSCLC CT scans; p-value is 0.000e+00.

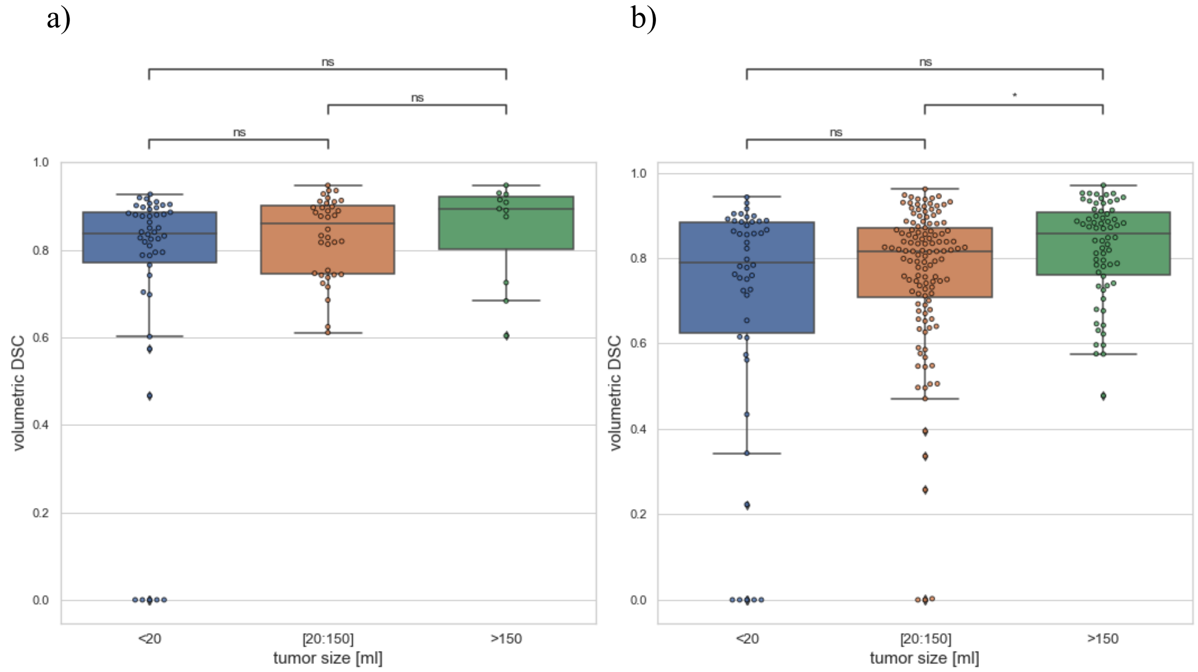

Fig. 6: Quantitative segmentation performance in regards to tumor size category. Quantitative segmentation performance is measured with volumetric dice similarity coefficient (DSC). Data are presented as box plots with overlaid swarm plot, where boxes are representing inter quartile range (IQR), extending from Q1 to Q3 and centered on the median value. Upper whiskers represent the highest data point that is less than Q3 + 1.5 × IQR. Lower whiskers represent the smallest data point that is greater than Q1 − 1.5 × IQR. Data points outside whiskers considered as outliers. P-values were calculated using a two-sided Mann-Whitney-Wilcoxon test with Bonferroni correction and referred as follows: “ns” on the plot refers to the p-value in the range: 5.00e-02 < p <= 1.00e+00; * refers to the p-value in the range: 1.00e-02 < p <= 5.00e-02. The exact p values reported in the order from the top to the bottom as they displayed on the figures. **a)** test dataset containing 93 NSCLC independent CT scans; corresponding p-values: 1.758e-01, 1.000e+00, 7.804e-01. **b)** external validation dataset of 236 independent NSCLC CT scans; corresponding p-values: 5.185e-02, 4.180e-02, 1.000e+00.

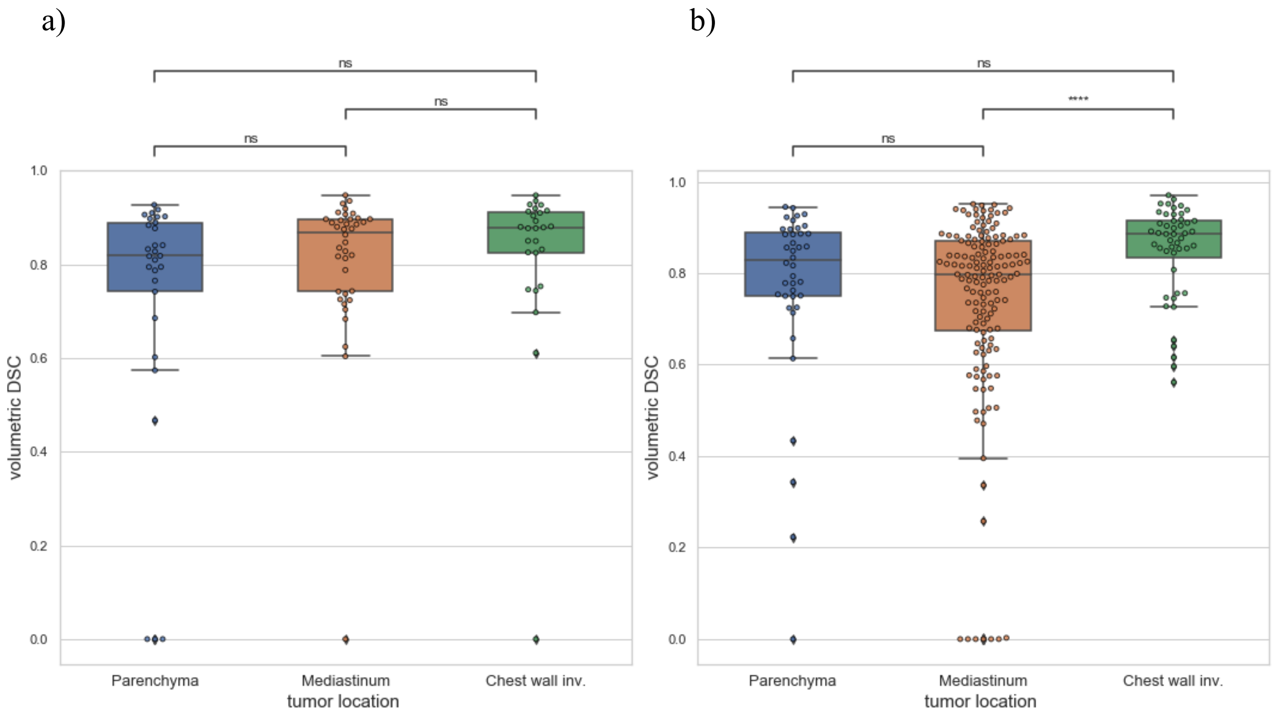

Fig. 7: Quantitative segmentation performance in regards to the tumor location. Quantitative segmentation performance is measured with volumetric dice similarity coefficient (DSC). Data are presented as box plots with overlaid swarm plot, where boxes are representing inter quartile range (IQR), extending from Q1 to Q3 and centered on the median value. Upper whiskers represent the highest data point that is less than Q3 + 1.5 × IQR. Lower whiskers represent the smallest data point that is greater than Q1 − 1.5 × IQR. Data points outside whiskers considered as outliers. P-values were calculated using a two-sided Mann-Whitney-Wilcoxon test with Bonferroni correction and referred as follows: "ns" on the plot refers to the p-value in the range: 5.00e-02 < p <= 1.00e+00; **** refers to the p-value in the range: p <= 1.00e-04. The exact p values reported in the order from the top to the bottom as they displayed on the figures. **a)** test dataset containing 93 NSCLC independent CT scans; corresponding p-values: 1.514e-01, 1.000e+00, 7.800e-01. **b)** external validation dataset of 236 independent NSCLC CT scans; corresponding p-values: 6.150e-02, 2.161e-05, 4.203e-01.

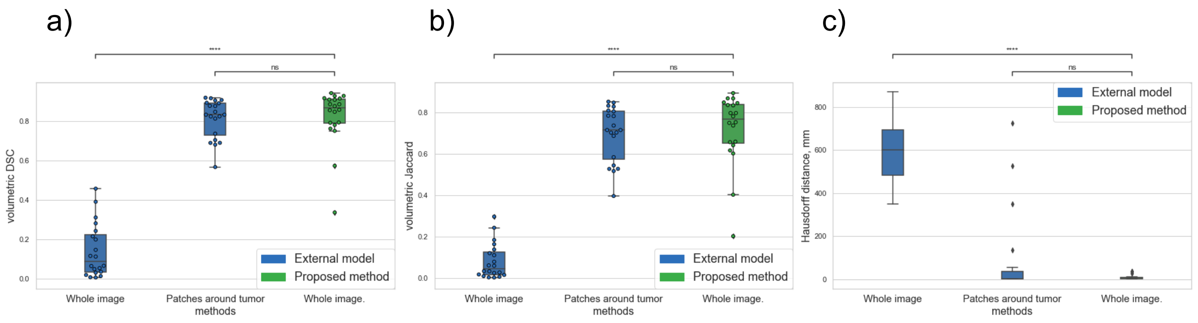

Fig. 8:* Comparison to a published method**. Comparison to a published automatic segmentation method based on the external validation dataset containing 20 independent NSCLC CT scans. Data are presented as box plots with overlaid swarm plot, where boxes are representing inter quartile range (IQR), extending from Q1 to Q3 and centered on the median value. Upper whiskers represent the highest data point that is less than $Q3 + 1.5 \times IQR$. Lower whiskers represent the smallest data point that is greater than $Q1 - 1.5 \times IQR$. Data points outside whiskers considered as outliers. P-values were calculated using a two-sided Mann-Whitney-Wilcoxon test with Bonferroni correction and referred as follows: “ns” on the plot refers to the p-value in the range: 5.00e-02 < p <= 1.00e+00; * refers to the p-value in the range: p <= 1.00e-04. The exact p values reported in the order from the top to the bottom as they displayed on the figures. **a)** comparison of volumetric dice similarity coefficient (DSC); corresponding p-values: 1.835e-07, 5.706e-01. **b)** comparison of volumetric Jaccard index; corresponding p-values: 1.835e-07, 5.706e-01. **c)** comparison of $Hausdorff_{95th}$ distance; corresponding p-values: 1.284e-07, 1.000e+00.

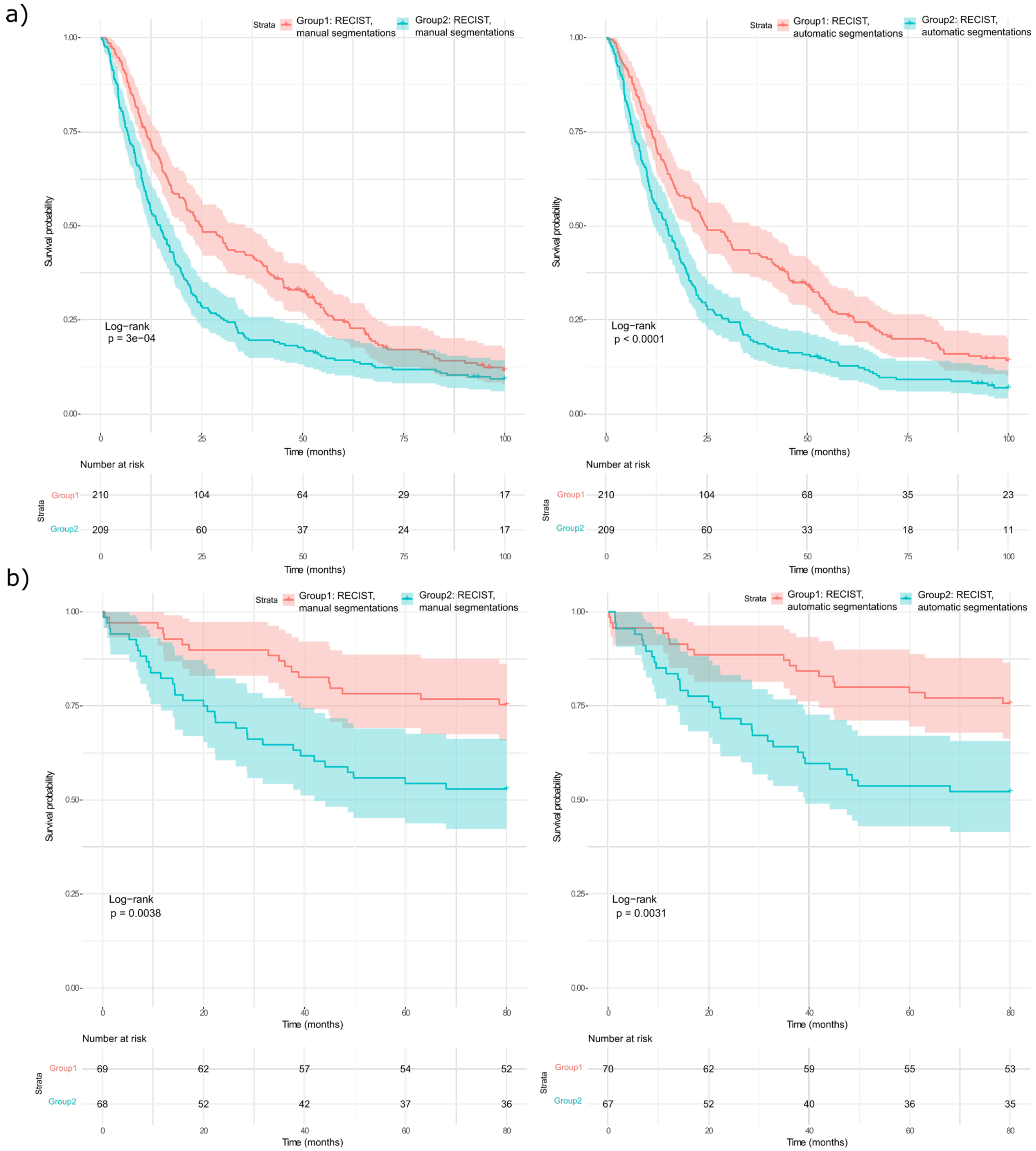

Fig. 9: Prognostic power of NSCLC segmentations measured with RECIST. Prognostic power of Non-Small Cell Lung Cancer (NSCLC) segmentations is measured through RECIST biggest diameter. RECIST measurement is calculated based on the manual and automatically generated contours. Kaplan Meyer curves for survival groups based on RECIST displayed with 95% pointwise confidence intervals. P-values are calculated using the log-rank test. Vertical hash marks indicate censored data. **a)** Maastro-CT-Lung-1 cohort of 419 NSCLC patients. **b)** Stanford Lung cohort of 137 NSCLC patients.

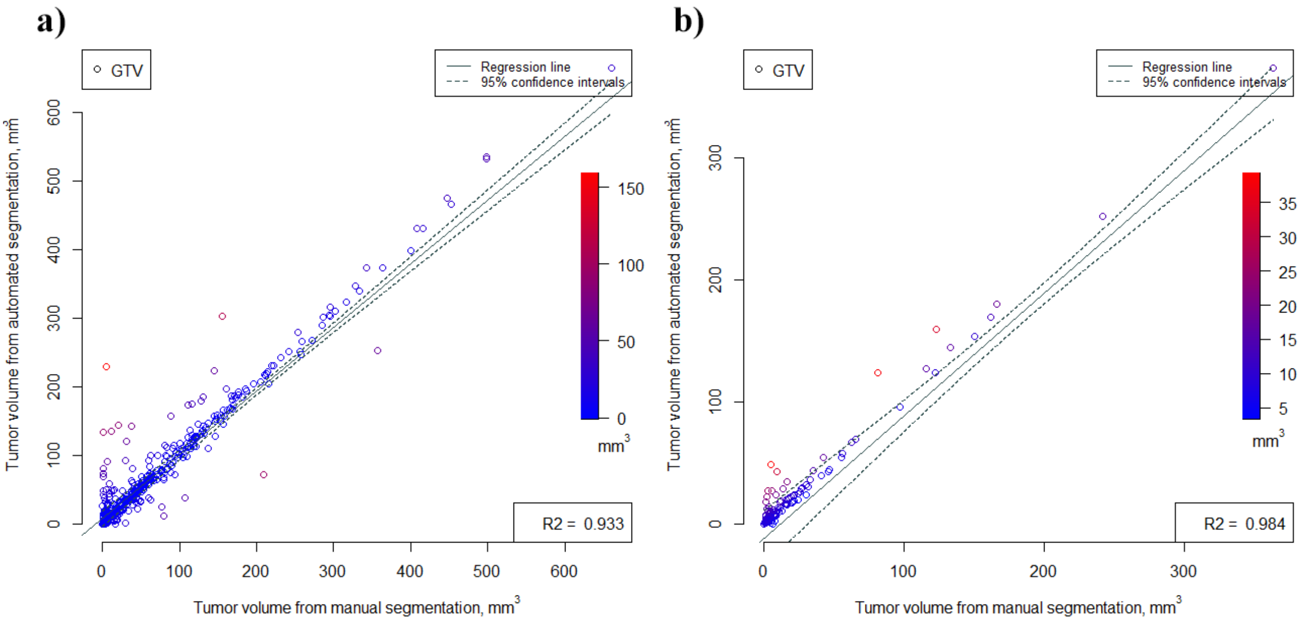

***Fig. 10:* Scatter plots for tumor volume**. Scatter plots for automated vs manual tumor volume (GTV) with the color map that highlights the distance from the regression line in mm3. **a)** Maastro-CT-Lung-1 cohort of 419 NSCLC patients, **b)** Stanford Lung cohort of 137 NSCLC patients.

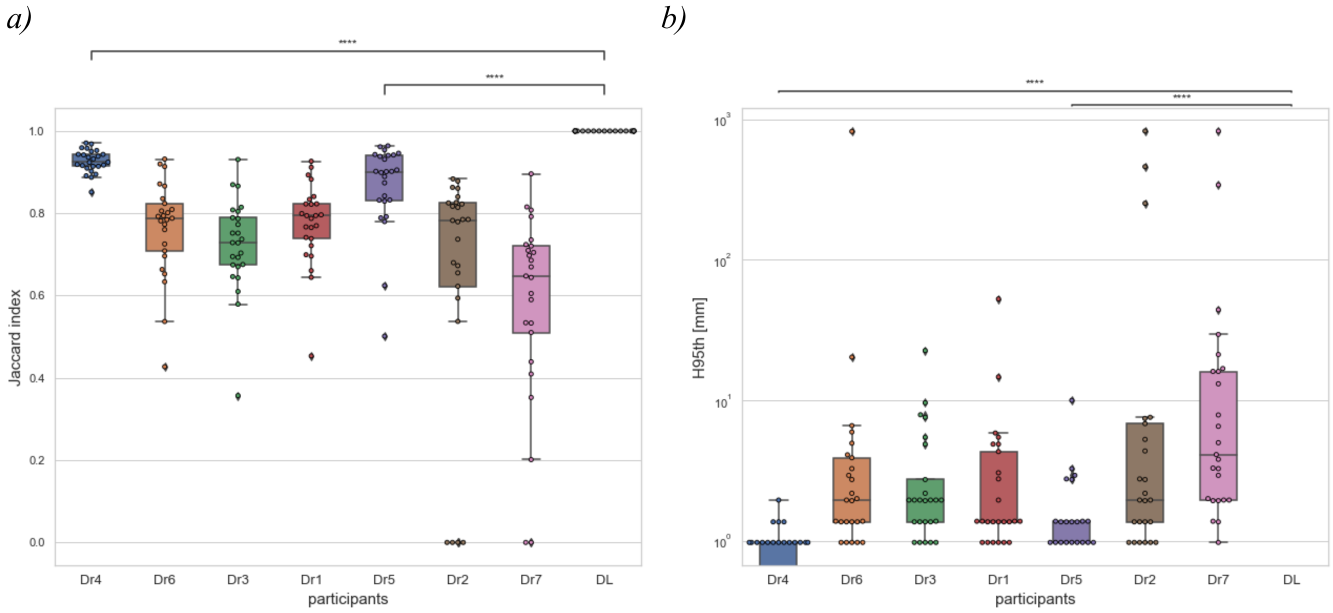

***Fig.* 11**: **Intra-observer variability.** Intra-observer variability measured in the in-silico clinical trial setting across 7 participant's (DR1-DR7) and proposed method (DL). Intra-observer variability measured on the 25 independent NSCLC CT segmentations. Data are presented as box plots with overlaid swarm plot, where boxes are representing inter quartile range (IQR), extending from Q1 to Q3 and centered on the median value. Upper whiskers represent the highest data point that is less than Q3 + 1.5 × IQR. Lower whiskers represent the smallest data point that is greater than Q1 − 1.5 × IQR. Data points outside whiskers

considered outliers. P-values were calculated using a two-sided Mann-Whitney-Wilcoxon test with Bonferroni correction and referred as follows: **** refers to the p-value in the range: p <= 1.00e-04. The exact p values reported in the order from the top to the bottom as they displayed on the figures. Dr1, Dr2, Dr3, Dr4, Dr5, Dr6, Dr7 - represent contours made by the medical doctors, DL -represents automatically generated contours. **a)** Volumetric Jaccard index representing intra-observer variability, across participants and the automated method, obtained on the 25 NSCLC patients by seven participants and the automated method; corresponding p values are 1.946e-10, 1.946e-10. **b)** Robust Hausdorff distance (H95th) for intra-observer variability, across participants and the automated method, obtained on the 25 NSCLC patients, corresponding p values are: 6.250e+02, 6.250e+02.

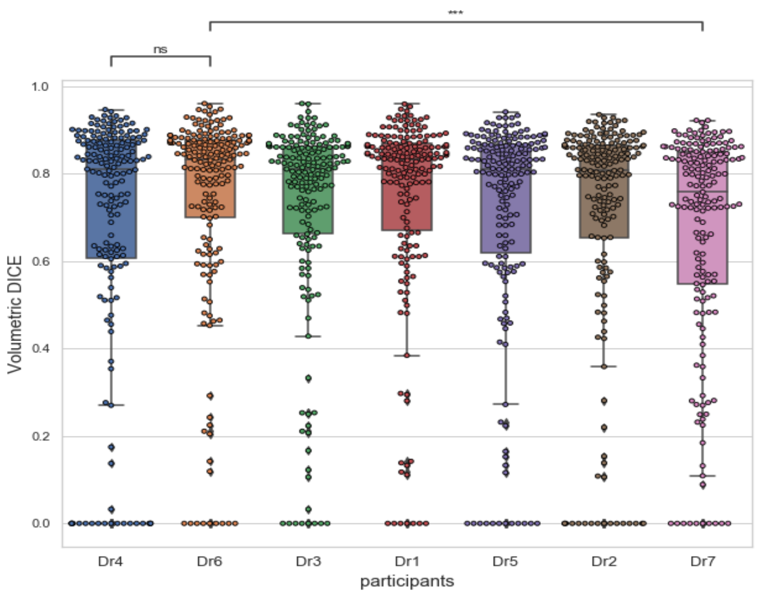

***Fig.* 12: Inter-observer variability.** Distributions of participant's (DR1-DR7) inter-observer variability measured on 25 independent NSCLC CT scan segmentations using volumetric dice. Data are presented as box plots with overlaid swarm plot, where boxes are representing inter quartile range (IQR), extending from Q1 to Q3 and centered on the median value. Upper whiskers represent the highest data point that is less than Q3 + 1.5 × IQR. Lower whiskers represent the smallest data point that is greater than Q1 − 1.5 × IQR. Data points outside whiskers considered outliers. P-values were calculated using a two-sided Mann-Whitney-Wilcoxon test with Bonferroni correction and referred as follows: "ns" on the plot refers to the p-value in the range: 5.00e-02 < p <= 1.00e+00; *** refers to the p-value in the range: 1.00e-04 < p <= 1.00e-03. The exact p values reported in the order from the top to the bottom as they displayed on the figures. Dr1, Dr2, Dr3, Dr4, Dr5, Dr6, Dr7 - represent contours made by the medical doctors. Corresponding p values are 1.705e-04, 2.842e-01.

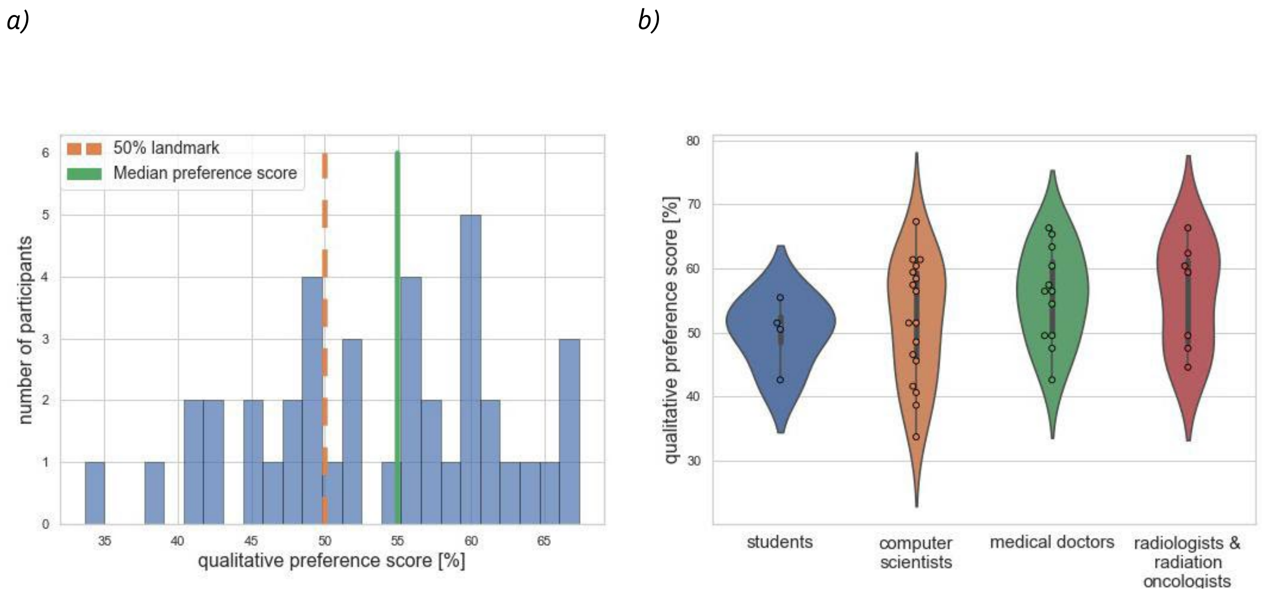

Fig. 13: In-silico clinical trial, qualitative method performance.

Qualitative segmentation performance measured with preference score on 40 participants: **a)** distribution of the scores for all participants, **b)** grouped scores

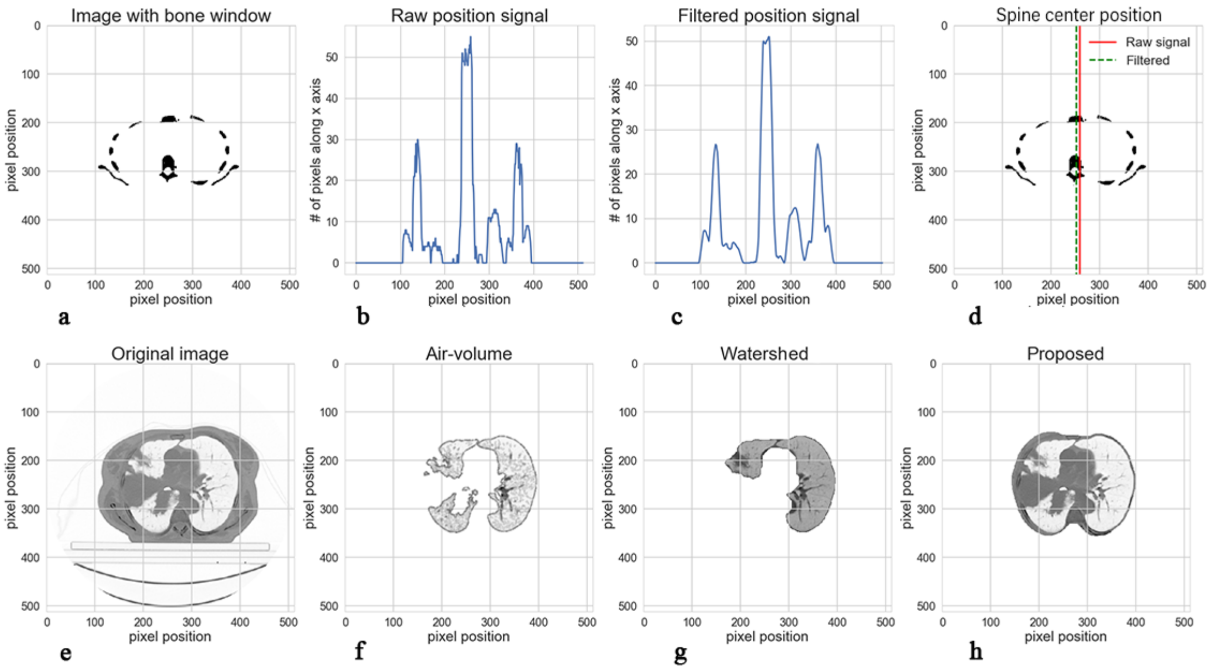

Fig. 14: Visualization of lung region isolation steps. **a)** Original image with bone window settings (WW:1800, WL:400); **b)** Sum of image slice intensity values projected onto the coronal plane; **c)** Same as **b)** with a 7th order moving average filter applied; **d)** Center of the spine found by using **b)**- red line, and by using **c)** - green dashed line; **e)** Original dicom slice;

f) Lung mask obtained using air-filled connected volumes for comparison purposes[1]; **g)** Lung mask obtained using watershed algorithm for comparison purposes [2]; **h)** Lung mask obtained using proposed lung region isolation algorithm.

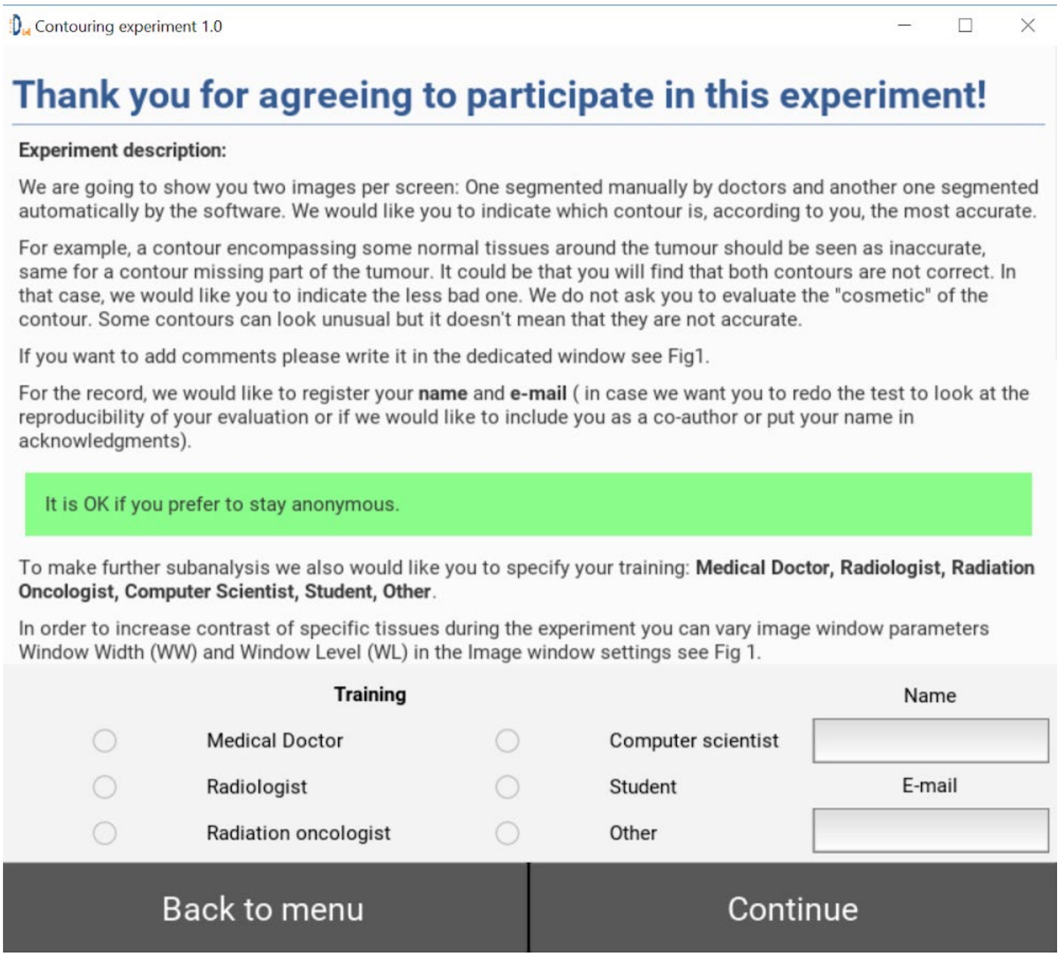

Fig. 15: In-silico clinical trial, qualitative assessment software's intro screen. Qualitative assessment screen with a description of the experiment and a small questionnaire.

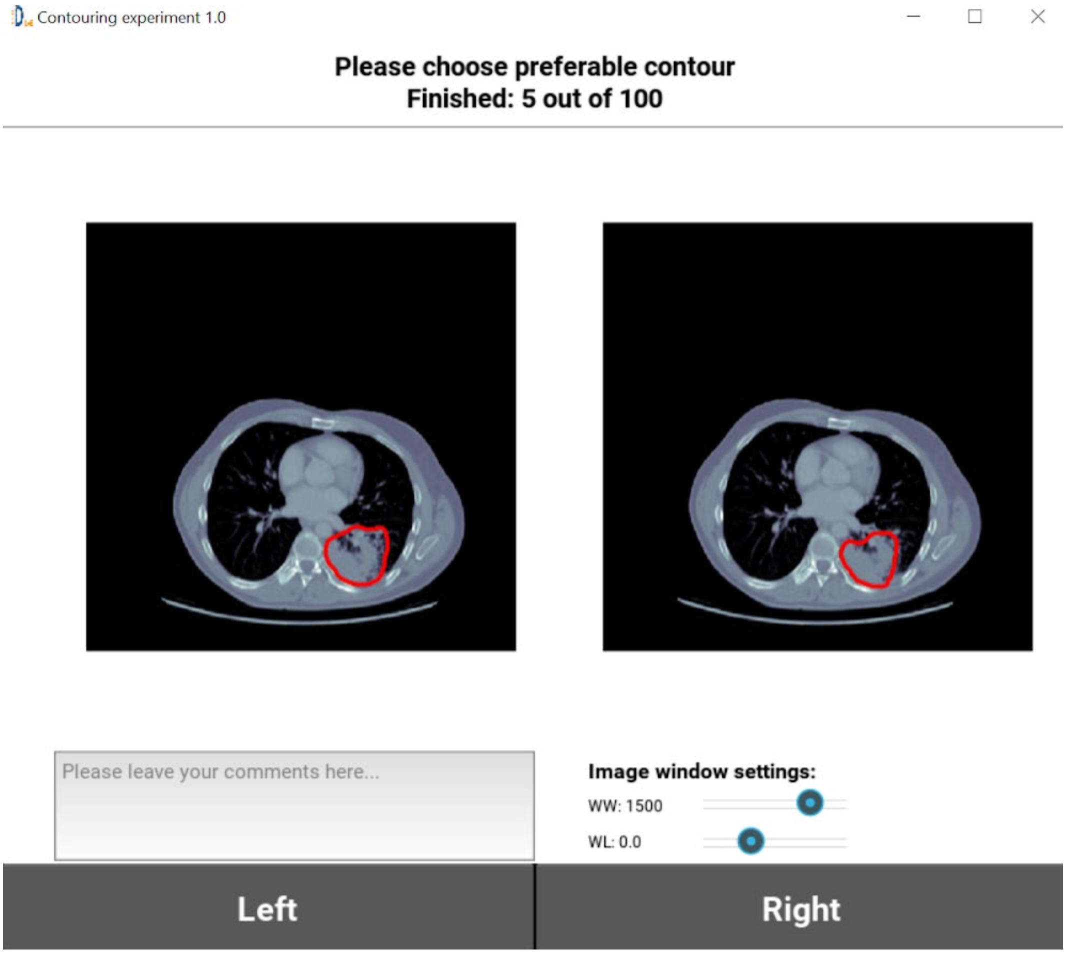

Fig. 16: In-silico clinical trial, qualitative assessment software's assesment screen. Screenshot of the qualitative assessments screen. Image window settings allows for window filtering, where WW refers to Window Width and WL to Window Level in Hounsfield scale.

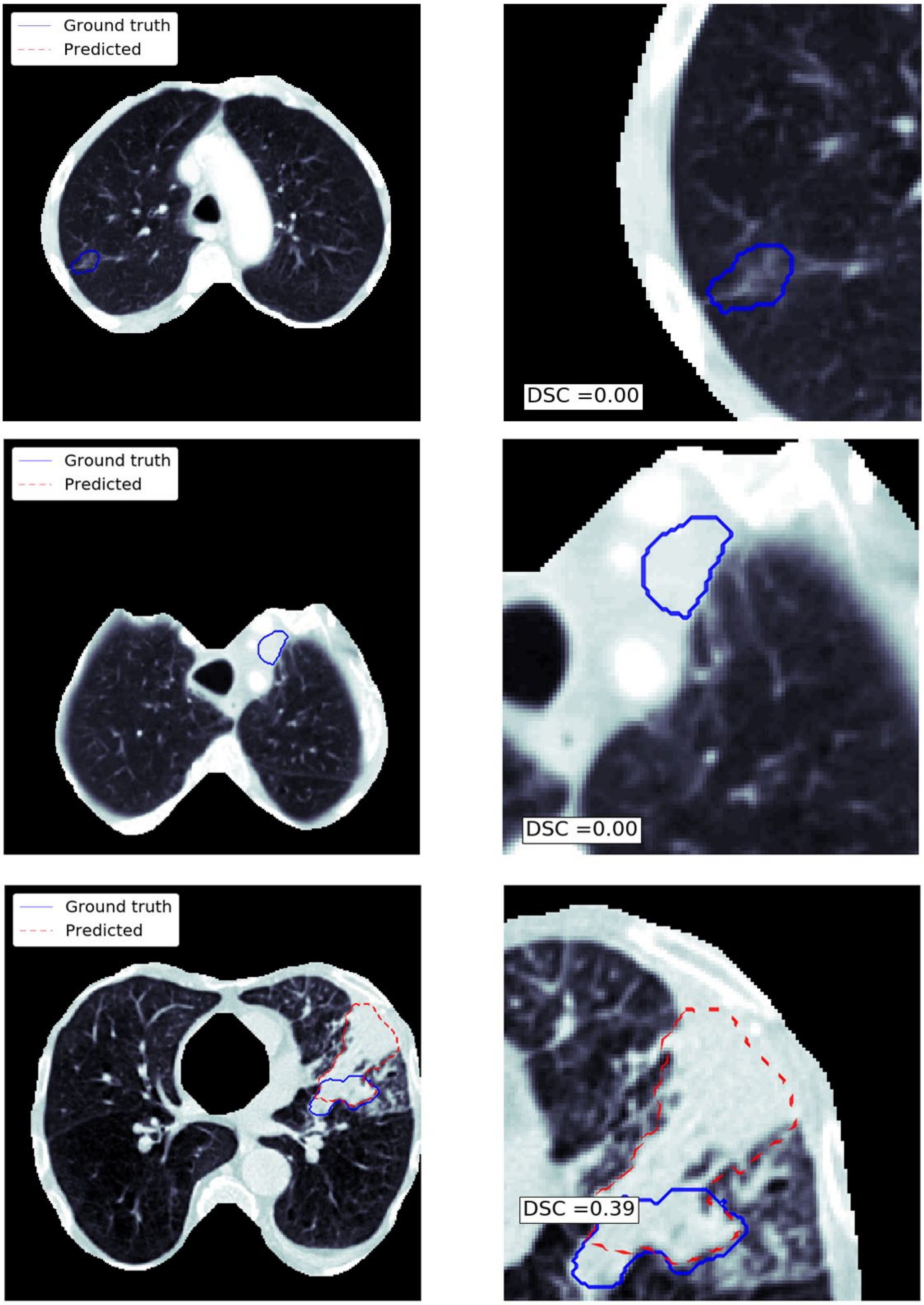

Fig. 17: Examples of failed segmentations. Some of the cases where segmentation failed. Automatically generated tumor segmentations are shown as red lines while manual segmentations are shown in blue. Second column displays magnified area around the tumor. Corresponding 2D dice similarity coefficient is provided in the bottom left corner.

References

1. Zhou, S., Cheng, Y. & Tamura, S. Automated lung segmentation and smoothing techniques for inclusion of juxtapleural nodules and pulmonary vessels on chest CT images. Biomed. Signal Process. Control 13, 62–70 (2014).

2. Shojaii, R., Alirezaie, J. & Babyn, P. Automatic lung segmentation in CT images using watershed transform. in IEEE International Conference on Image Processing 2005 vol. 2 II–1270 (2005).
