# Supplementary material for: Automated detection and segmentation of non-small cell lung cancer computed tomography images
Source: Nat Commun. 2022 Jun 14;13:3423. doi: 10.1038/s41467-022-30841-3 (PMC9198097; doi:10.1038/s41467-022-30841-3)
Supplement: Supplementary file 3 — Reporting Summary [file 41467_2022_30841_MOESM3_ESM.pdf]

nature research

Corresponding author(s): Philippe Lambin, PHD

Last updated by author(s): May 9, 2022

Reporting Summary

Nature Research wishes to improve the reproducibility of the work that we publish. This form provides structure for consistency and transparency in reporting. For further information on Nature Research policies, see our Editorial Policies and the Editorial Policy Checklist.

Statistics

For all statistical analyses, confirm that the following items are present in the figure legend, table legend, main text, or Methods section.

n/a	Confirmed	
[ ]	[x]	The exact sample size (*n*) for each experimental group/condition, given as a discrete number and unit of measurement
[x]	[ ]	A statement on whether measurements were taken from distinct samples or whether the same sample was measured repeatedly
[ ]	[x]	The statistical test(s) used AND whether they are one- or two-sided<br>*Only common tests should be described solely by name; describe more complex techniques in the Methods section.*
[x]	[ ]	A description of all covariates tested
[x]	[ ]	A description of any assumptions or corrections, such as tests of normality and adjustment for multiple comparisons
[ ]	[x]	A full description of the statistical parameters including central tendency (e.g. means) or other basic estimates (e.g. regression coefficient) AND variation (e.g. standard deviation) or associated estimates of uncertainty (e.g. confidence intervals)
[ ]	[x]	For null hypothesis testing, the test statistic (e.g. *F*, *t*, *r*) with confidence intervals, effect sizes, degrees of freedom and *P* value noted<br>*Give P values as exact values whenever suitable.*
[x]	[ ]	For Bayesian analysis, information on the choice of priors and Markov chain Monte Carlo settings
[x]	[ ]	For hierarchical and complex designs, identification of the appropriate level for tests and full reporting of outcomes
[x]	[ ]	Estimates of effect sizes (e.g. Cohen's *d*, Pearson's *r*), indicating how they were calculated

Our web collection on statistics for biologists contains articles on many of the points above.

Software and code

Policy information about availability of computer code

Data collection	The data was collected in the DICOM format using private and open sources as described in the Data Availability section of the original article.
Data analysis	Sofware used:<br>Pre-processing, data handling: Python 3.7 (os, re, random, time, psutil 5.6.1, numpy 1.16.2, pandas 0.25.1, pydicom 1.3.0, opencv-python 4.1.0.25, SimpleITK 1.2.0, scikit-image 0.14.2, scipy 1.2.1, scikit-learn 0.23.2, tqdm 4.40.2)<br>Deep learning: Python 3.7 (tensorflow-gpu 1.15.0, keras 2.2.4, livelossplot 0.4.1)<br>Visualization: Python 3.7 (matplotlib 3.0.3, seaborn 0.9.0)<br>Survival analysis: R (version 4.0.2) packages: survival (version 3.1-12) and survminer (version 0.4.7)<br>Conversion of DICOM to NRRD: Precision medicine toolbox (https://github.com/primakov/precision-medicine-toolbox)<br>The code and model files are provided in the project GitHub repository: https://github.com/primakov/DuneAI-Automated-detection-and-segmentation-of-non-small-cell-lung-cancer-computed-tomography-images

For manuscripts utilizing custom algorithms or software that are central to the research but not yet described in published literature, software must be made available to editors and reviewers. We strongly encourage code deposition in a community repository (e.g. GitHub). See the Nature Research guidelines for submitting code & software for further information.

Data

Policy information about availability of data

All manuscripts must include a data availability statement. This statement should provide the following information, where applicable:

- Accession codes, unique identifiers, or web links for publicly available datasets
- A list of figures that have associated raw data
- A description of any restrictions on data availability

All the open source data used in the article has the appropriate references. The processed datasets 2,3,4,5,9,10 are available under restricted access as they were provided under Data Transfer Agreements from corresponding centers, and are not yet public due to data privacy laws, access can be obtained through the corresponding author upon request subject to ethical review. Approximate time for processing the data request is one month. Although raw CT data for datasets 2,3,4,5,9,10 can not be shared, all measured results to reproduce the statistical analysis are shared on the GitHub repository (https://github.com/primakov/DuneAI-Automated-detection-and-segmentation-of-non-small-cell-lung-cancer-computed-tomography-images). We are also providing the publicly available minimum dataset with deep-learning and manual segmentations. Additionally, we can provide method's segmentations and manual segmentations for the test/ validation datasets upon request from the corresponding author.

Field-specific reporting

Please select the one below that is the best fit for your research. If you are not sure, read the appropriate sections before making your selection.

- [x] Life sciences
- [ ] Behavioural & social sciences
- [ ] Ecological, evolutionary & environmental sciences

For a reference copy of the document with all sections, see nature.com/documents/nr-reporting-summary-flat.pdf

Life sciences study design

All studies must disclose on these points even when the disclosure is negative.

Sample size	The sample size used in the study was determined by the amount of the available open source data and amount of data available through DTAs. 1328 CT scans in total (90 562 unique axial CT slices), training & testing on 1105 CT scans (64 896 unique CT slices) + augmented data, Validation 238 CT scans (25 666) unique CT slices.
Data exclusions	3 patients from Dataset 1, 1 patient from Dataset 4, 9 patients from Dataset 7, 2 patients from Dataset 8, were excluded due to missing tumor contour and/or lack of pet scan to perform the segmentations according to clinical protocol.
Replication	All measured results to replicate statistical analysis and figures reported in this manuscript are shared. Additionally, we provide model and model weights and method's segmentations for the minimum dataset. Segmentations for the test and validation datasets (manual& automatic) can be provided by request from the corresponding author. Results of this manuscript have been replicated to ensure the correctness.
Randomization	The randomization was applied for splitting into training/testing cohorts (described in the methods section), and for gathering the experts preference score for clinical trial.
Blinding	Blinding was used in in silico clinical trial for gathering the response from the participants of qualitative evaluation.

Reporting for specific materials, systems and methods

We require information from authors about some types of materials, experimental systems and methods used in many studies. Here, indicate whether each material, system or method listed is relevant to your study. If you are not sure if a list item applies to your research, read the appropriate section before selecting a response.

Materials & experimental systems

n/a	Involved in the study	
x		Antibodies
x		Eukaryotic cell lines
x		Palaeontology and archaeology
x		Animals and other organisms
	x	Human research participants
	x	Clinical data
x		Dual use research of concern

Methods

n/a	Involved in the study	
x		ChIP-seq
x		Flow cytometry
x		MRI-based neuroimaging

Human research participants

Policy information about studies involving human research participants

Population characteristics	Patients characteristics are reported in the supplementary table 2.
Recruitment	Retrospective data collection
Ethics oversight	All methods were carried out in accordance with the relevant guidelines and regulations.The institutional review board of Maastricht University Medical Center has waived the need for informed consent, since the data was anonymized and retrospectively collected with no intervention planned for participants based on the study, and no compensations were provided.

Note that full information on the approval of the study protocol must also be provided in the manuscript.

Clinical data

Policy information about clinical studies

All manuscripts should comply with the ICMJEguidelines for publication of clinical research and a completedCONSORT checklist must be included with all submissions.

Clinical trial registration	NCT04164186
Study protocol	https://clinicaltrials.gov/ct2/show/NCT04164186?term=Segmentation&draw=2&rank=1
Data collection	The data was publicly available from the mentioned centers and the clinical data was provided along with the images. Please see Table1. Data were collected retrospectively from the open source.
Outcomes	Survival time in days from the moment of scan to the time of the event, with right censoring
